# Editorial: Neuronal ceroid lipofuscinosis: molecular genetics and epigenetics

**DOI:** 10.3389/fgene.2024.1430703

**Published:** 2024-06-26

**Authors:** Paschalis Nicolaou

**Affiliations:** The Cyprus Institute of Neurology and Genetics, Nicosia, Cyprus

**Keywords:** neuronal ceroid lipofuscinosis, batten disease, NCLs, lysosomal storage disorders, molecular genetics, epigenetics

The neuronal ceroid lipofuscinoses (NCLs), also known as Batten disease is a group of inherited neurodegenerative disorders that are characterised by motor impairment, cognitive decline, progressive, epilepsy, ataxia and early death. NCLs are clinically classified into four major types based on the age of onset of the disease, infantile (6–24 months), late-infantile (2–4 years), juvenile (5–10 years), and adult-onset (>18 years). NCLs according to the associated gene classified into different subtypes. Different subtypes of N*CL* disease are caused by mutations in thirteen genes (*CLN1*-*8* and *10*–*14*), which results in substantial clinical variation, including symptoms and age of onset. The prevalence of the disease is 8–10 per 100,000 individuals. NCLs are characterized by the accumulation of auto-fluorescence lipopigments in various tissues and cell types. The majority of NCLs are inherited in an autosomal recessive manner, however, autosomal dominant inheritance has been reported in one CLN4adult-onset disease. Patients with NCL have shortened life expectancy depending on the type of NCL that patient has.

Next-generation sequencing has revolutionized the field of medical genetics allowing us the identification of disease-causing variants. This is essential for improving our understanding of disease pathogenesis, mechanisms and pathways. In addition transcriptomics, proteomics and epigenomics analysis will lead to the identification of disease biomarkers. This knowledge will facilitate the development of personalised diagnostic and therapeutic approaches.

CLN proteins have diverse cellular roles related to autophagy, signal transduction, lipid homeostasis, lysosomal ion homeostasis, and intracellular trafficking The ceroid lipofuscinoses neuronal *(CLN)* genes encode proteins that are found mostly in the endoplasmic reticulum (ER), endosomes or lysosomes. *PPT1*/CLN1, *TPP1*/CLN2, *CLN5/*CLN5, *CTSD*/CLN10, *GRN*/CLN11 and *CTSF*/CLN13 are lysosomal proteins that also localize extracellularly, CLN6, CLN8 proteins that localize to the endoplasmic reticulum (ER). *DNAJC5*/CLN4 and *KCTD7*/CLN14 are peripherally associated membrane proteins. CLN3, *MFSD8*/CLN7, and *ATP13A2*/CLN12 are transmembrane proteins.

This Research Topic aims to provide novel evidence and up-to-date high-impact knowledge and review existing data on the medical and molecular translational research in NCLs leading to an inclusive understanding of etiology and pathogenesis of the disease. The Topic includes four original research papers.

CLN5 is implicated in several cellular processes including endosomal sorting, biometal homeostasis, sphingolipid metabolism, and autophagy. However, the exact role of CLN5 in the cell is not well known.

Kim et al. investigated the effect of *cl5* (*CLN5* Human Homologues) -deficiency on *Dictyostelium* during growth and development. They used transcriptomics to detect genes that are affected by cln5 -deficiency in *D. discoideum* during growth and starvation. Through this study, they discover the genes, proteins, and enzymes affected by cln5-deficiency in *D. discoideum* and provide insight into the pathways affected by *CLN5* pathogenic variants in humans. They identified differentially expressed genes that are associated with proteasomal degradation in *cln5*.

Murray et al. evaluated dual intracerebroventricular (ICV) and intravitreal (IVT) administration of a self-complementary adeno-associated viral vector encoding ovine Aries *CLN5* (scAAV9/o*CLN5*) into CLN5 affected and presymptomatic sheep (*CLN5* ^*−/−*^) and at various disease stages and various treatment doseges. Increased CLN5 protein expression was detected throughout the brain and spinal cord, and improvements in the central nervous system and retinal disease relate were observed. These findings provide evidence for improving the quality of life in *CLN5* deficient patients and encourage the initiation of clinical trials in *CLN5* deficient patients and presymptomatic individuals.

Mitchell et al. present the results of a large study of AAV therapy for *CLN5* deficiency in an ovine Aries model. The authors describe a pre-clinical study of intracerebroventricular (ICV) gene therapy as a therapeutic modality for a CLN5 neurodegenerative lysosomal storage disorder. The study described the use of a novel self-complementary AAV9 virus to deliver the ovine Aries *CLN5* gene in a previously established ovine Aries model of CLN5 disease. A naturally occurring sheep model was used to validate the efficacy of the new treatment at pre-symptomatic, early symptomatic, and advanced symptomatic stages of the disease. They showed that the treatment delayed disease progression and was able to amend different features of the disease, including lysosomal storage and inflammation and improved the CLN5 brain distribution with delivery scAAV9/oCLN5 at three stages of the disease.

The CLN3 protein participates in many compartments within cells. CLN3 is localized to the lysosome, which are cellular compartments that digest and recycle different types of molecules and are necessary for lysosomal function. The age of onset is usually around 5 years old and the disease progresses rapidly with premature death around 20 years of age. CLN3 is characterised by vision loss, seizures and motor, cognitive, and behavioural deficits.

Johnson et al. present a preclinical study of an AAV9 gene therapy for CLN3 disease. They used a mouse model to test the most common mutation found in human CLN3 disease patients. They delivered an AAV9 vector encoding human *CLN3* via a single intracerebroventricular injection and provided strong evidence for the utility of such a strategy in the treatment of patients with CLN3 disease. The results are encouraging and give the potential for future clinical trials.

In conclusion, the scientific articles included in this Research Topic provide novel knowledge of inherited Neuronal Ceroid Lipofuscinosis. This knowledge will enable the understanding of the underlying mechanisms and facilitate the development of targeted diagnostic and personalized therapeutic approaches.

